# A mindset of competition versus cooperation moderates the impact of social comparison on self-evaluation

**DOI:** 10.3389/fpsyg.2015.01337

**Published:** 2015-09-03

**Authors:** Lucie Colpaert, Dominique Muller, Marie-Pierre Fayant, Fabrizio Butera

**Affiliations:** ^1^Department of Psychology, University of GenevaGeneva, Switzerland; ^2^Department of Psychology, University Grenoble Alpes, GrenobleFrance; ^3^Institut Universitaire de France, ParisFrance; ^4^Department of Psychology, University of LausanneLausanne, Switzerland

**Keywords:** social comparison, cooperation, competition, mindset, self-evaluation

## Abstract

Do people feel better or worse about themselves when working with someone who is better than they are? We present the first replication of the work of Stapel and Koomen (2005), who showed that being in a competitive vs. cooperative mindset moderates the effects of social comparison on self-evaluation. In Experiment 1, we present a close replication of Stapel and Koomen (2005, Study 2). Participants in competition/cooperation had to self-evaluate after receiving information about the personal characteristics of an upward/downward comparison target. In Experiment 2, we went further by providing feedback about both the comparison target and the self. Our results and a small-scale meta-analysis combining our experiments and Stapel and Koomen’s (2005) confirm that a competitive/cooperative mindset moderates the impact of social comparison on self-evaluation; nevertheless, the effect size we found across the two experiments is clearly more modest than the one found in Stapel and Koomen’s (2005) work.

## Introduction

Do people feel better or worse about themselves when working with someone who is better than they are? This question refers to a vast body of literature on social comparison showing that comparing oneself to a superior (as opposed to an inferior) other can be inspiring, but can also lead people to feel bad about themselves (e.g., Muller and Fayant, 2010). In this article, we present the first replication of the work of Stapel and Koomen (2005), who showed that being in a competitive vs. cooperative mindset moderates the effects of social comparison on self-evaluation, and discuss similarities and differences between our findings and theirs.

One can feel worse after comparing with someone who is better (i.e., an upward comparison) than after comparing with someone who is worse than oneself (i.e., a downward comparison; Morse and Gergen, 1970). The literature on social comparison has described this phenomenon as a contrast effect. However, one can also feel better after an upward than a downward comparison, which the literature refers to as an assimilation effect. Previous work has identified several determinants of contrast and assimilation effects. For instance, a sense of similarity orients the effect of social comparison from contrast to assimilation (Mussweiler, 2003). Experiential cues (e.g., walking while processing comparison information) that one is moving toward the target instead of away from her/him also produce a switch from contrast to assimilation (Fayant et al., 2011). Critically for the current research, a sense of communality with the target, like sharing group membership, sharing a birthday, or an interdependent self-construal, also causes a shift from contrast to assimilation (Brown et al., 1992; Brewer and Weber, 1994; Kemmelmeier and Oyserman, 2001). In sum, contrast and assimilation are both likely to occur in the course of social comparison under different circumstances.

The Inclusion/Exclusion Model is one of the most comprehensive models explaining whether contrast or assimilation would occur (Schwarz and Bless, 2007; Bless and Schwarz, 2010). This model can be applied to self-evaluation after social comparison and more generally to judgments of any kind. The general question of this model is what happens when a piece of information (for instance, in the current context, information about a comparison target) is made available before a judgment (for instance, in the current context, an evaluation of the self). This model predicts that the influence of this piece of information depends on how it is used, namely, either as a standard against which the object of judgment is evaluated or as information that is included in the representation of the object of judgment. When this piece of information is used as a standard, this model predicts a contrast effect: the more positive (negative) the information is, the less positive (negative) the judgments about the object are. When this piece of information is included in the representation of the object of judgment, this model predicts an assimilation effect: the more positive (negative) this information is, the more positive (negative) the judgments about the object are. Applied to a social comparison context, this means that, when judging the self, information about a comparison target will lead to a contrast effect when it is used as a standard, while this information will lead to an assimilation effect when it is included in the representation of the self.

Importantly, one variable that could orient toward excluding vs. including the comparison target in the representation of the self is a competitive vs. cooperative mindset (Schwarz and Bless, 2007). Two reasons lead us to expect a moderating role of these mindsets. First, in competition, by definition at least two units are salient—the self and the competing other(s)—whereas cooperation imposes a shared category which fosters the inclusion of the self and the comparison other into the same unit (Schwarz and Bless, 2007). Therefore, exclusion from the self (i.e., use of the comparison target as a standard) should be more likely in competition and inclusion should be more likely in cooperation. Indirectly supporting this reasoning, previous research showed that a sense of communality moderates the effect of social comparison information on self-evaluation (e.g., Brown et al., 1992). Second, previous work showed that a conflict mindset (often seen as typical of competition) leads to less inclusiveness than a cooperative mindset (Carnevale and Probst, 1998). In sum, the Inclusion/Exclusion model supports the hypothesis that a competitive/cooperative mindset should moderate the impact of social comparison information on self-evaluation.

In line with this idea, Stapel and Koomen (2005) already showed that a competitive/cooperative mindset moderates the effect of social comparison on self-evaluation. This work, however, has never been replicated. We argue that it is important to conduct a replication study for at least three reasons. First, although replication is always important—even more so when no previous replication has been published—it seems even more relevant for a study conducted by Diederik Stapel. Indeed, Stapel is now sadly famous for having published numerous fraudulent datasets. The final investigation conducted by a panel of experts concluded that 62 of Stapel’s publications contained signs of fraud (Levelt, 2012). The Stapel and Koomen’s (2005) paper is not one of these publications, but because of the circumstances (and the fact that for good reasons strong evidence was required to conclude that a paper contained fraud), it seems even more relevant to try to replicate such an important finding. Second, even if our work replicates Stapel and Koomen’s (2005) work, it is important to provide a better estimate of its effect size, given the practical implications of such work, for instance, in school or in organizations (e.g., Lam et al., 2011). Third, Stapel and Koomen’s (2005) studies only tested this effect when providing a description about the personal characteristics of the comparison other (e.g., “bright,” “serious”…), without mentioning the self. This is a limitation with regard to both the literature and real life settings. On the one hand, in many areas of the social comparison literature, feedback is provided for both the comparison target and the self (e.g., Buckingham and Alicke, 2002; Muller et al., 2004; Vancouver and Tischner, 2004; Muller and Butera, 2007; Quiamzade and Mugny, 2009; Normand and Croizet, 2013). Therefore, not providing feedback on the self is a methodological limitation for the study of social comparison and the generalization of such an effect. On the other hand, in real life settings, people often have access to and like to use information about the comparison target and about the self. Indeed, research has shown that students compare their own and others’ grades (Huguet et al., 2001), and that workers compare their own and others’ rejection/acceptance of a promotion (Schaubroek and Lam, 2004; Fischer et al., 2009) or wages (Gächter and Thöni, 2010).

In two experiments, we tested whether a competitive/cooperative mindset moderates the effect of social comparison on self-evaluation. In Experiment 1, we present a close replication of Stapel and Koomen (2005, Study 2) and provide only information about the comparison target. It is worth noting that among the studies presented in Stapel and Koomen’s (2005) article, we chose Study 2 because it was the experimental study with the most straightforward design. In Experiment 2, we use a paradigm where we provide (bogus) feedback about both the comparison target and the self. In both experiments, we test whether a competitive/cooperative mindset moderates the effect of social comparison on self-evaluation. Finally, we perform a meta-analysis combining Stapel and Koomen’s (2005) work and ours.

## Experiment 1

### Method

#### Participants

In line with Brandt et al.’s (2014) recommendations to use at least 2.5 times the original sample, we tripled Stapel and Koomen’s (2005, Study 2) sample size (*N* = 60) for the mindset by social comparison design (without the baseline condition). Accordingly, 198 psychology students (*N* = 165 women; *M*_age_ = 19.83, *SD*_age_ = 3.74) from a French university received extra course credit for what we presented as a “working group” study. We began collecting data in 2013, but because our comparison information referred to winter exams (making it impossible to collect data at another time of the year) and because we did not reach our expected sample size (*N* = 108), we also collected additional data a year later in 2014 (*N* = 90). Participants were randomly assigned to one of the four conditions of a 2 (direction of comparison: upward, downward^1^) × 2 (mindset: competition, cooperation) between-participants design. Because in France there is currently no law concerning non-interventional research, we did not seek explicit ethical approval for this research. All data were collected in accordance with the American Psychological Association’s ethical principles and analyzed anonymously.

#### Procedure

In Experiment 1, we followed Stapel and Koomen’s (2005) procedure as closely as possible, but made adjustments to reinforce the credibility of the cover story. These adjustments were the following: (a) we introduced the experiment as a study about working in groups (and did not specify the kind of task they had to perform until after the self-evaluation measure), (b) we did not promise participants extra credit to induce competitive mindset due to ethical concerns, (c) participants had to fill out a questionnaire before their lab appointment, (d) the description of the comparison target did not specify the sex of the target, (e) we ran the experiment just after the winter exam and before students got their grades.

We recruited participants at that time of the year because it helped to induce upward/downward comparison. Indeed, they were still unsure how they performed during these exams. Participants interested in taking part in the experiment received a short questionnaire with several questions (e.g., about the courses they had taken, their academic achievements, and their hobbies). To enhance the credibility of the target’s description (see below), they had to fill out this questionnaire and send it back before the beginning of the experiment. When participants registered, they were told that eight participants could participate at a time. Accordingly, we welcomed up to eight participants and assigned each of them to a computer.

All the instructions were displayed on the computer screen and informed the participants that we were interested in competition (cooperation) and that they would perform two tasks in which they would be in competition (cooperation) with one person in the room. Thus participants read that: “*Once these tasks are completed, we will be able to compute your score. Then your score will be compared (pooled) with the score of your opponent (teammate) to evaluate your performance.*” In other words, we manipulated structural competition and cooperation to the extent that our instructions created, respectively, negative and positive interdependence (Deutsch, 1949). It is also worth mentioning that with such a manipulation participants in the cooperation condition are told that their score will be computed and only then pooled with the other person’s score. In other words, in contrast to the manipulation used in the social loafing literature (e.g., Harkins and Szymanski, 1989), we did not minimize the possibility that participants felt possible for the experimenter to evaluate their individual output.

Next, we told participants that in such situations of competition (cooperation) it was important to have information about their opponent (teammate). This explained why they had to complete the short questionnaire they received by e-mail. At this point, the experimenter gave them a questionnaire where they read the alleged self-description of their opponent (teammate). In the upward comparison condition, the target wrote that he/she was very successful in school (both in high school and at the university), loved to read, had good social skills, and had many friends. In the downward comparison condition, the target wrote that he/she had difficulties at school, did not like to read, was rather lonely, and had only a few friends. We then told participants that, because some of their personal characteristics could have an impact on their score, we had to ask them a few more questions. Accordingly, participants then used 7-point scales (1 = *not at all* and 7 = *very*) to rate whether they felt they were successful in college, bright, competent, balanced, promising, and successful in life.

Following these self-evaluations questions, we measured whether participants thought their opponent (teammate) resembled them (1 = *not at all* and 7 = *very much*). Additionally, participants used 7-point scales (1 = *not at all* and 7 = *very*) to rate whether they felt their opponent (teammate) was intelligent, successful, and likable as manipulation checks of the direction of social comparison (see **Table 1A**, for bivariate correlations).

**Table 1:** Bivariate correlations between the measures (Panel A for Experiment 1 and Panel B for Experiment 2).

	Measure	1	2	3
**(A)**				
	(1) Self-evaluation	1	0.18*	0.27***
	(2) Similarity rating	0.18*	1	0.44***
	(3) Target evaluation	0.27***	0.44***	1
**(B)**				
	(1) Self-evaluation in visual tasks	1	0.34***	0.40***
	(2) Expected score	0.34***	1	0.26**
	(3) General self-evaluation	0.40***	0.26**	1

*p < 0.05; **p < 0.01; ***p < 0.001.

To be consistent with the cover story, participants then performed two tasks. The first was a cueing task (Muller and Butera, 2007) and the second was a cognitive reflection task (Frederick, 2005). Although the cueing task was also included in Experiment 2, these two cognitive tasks are not the focus of the current manuscript. Finally, after probing for suspicion, participants provided demographic information, were asked exploratory questions (whether they thought about the influence their partner’s performance could have on their own score and whether they thought this influence could be positive or negative on 7-point scales), and filled in a check for the mindset manipulation (“Please indicate if you were either in competition or in cooperation with the other person”). Because these exploratory questions did not reveal any significant effect, they will not be discussed further. Participants were carefully debriefed, thanked, and dismissed.

### Results

We conducted 2 (direction of comparison: upward, downward) × 2 (mindset: competition, cooperation) ANOVAs on the different measures. Below we also report 90% confidence intervals for the eta-squared (we used the 90% confidence intervals, because eta-squared cannot be negative and, therefore, 90% confidence intervals for eta-squared correspond to 95% confidence intervals for other indexes) and 95% confidence intervals for the Cohen’s *d* of pairwise comparisons.

#### Manipulation Check

We averaged the three items used to rate the target (α = 0.86). As could be expected, participants rated the upward comparison target more positively (*M* = 5.67, *SD* = 0.89) than the downward comparison target (*M* = 3.88, *SD* = 0.98), *F*(1,194) = 176.26, *MSE* = 0.88, *p* < 0.001, $\eta_{p}^{2}$ = 0.48, 90% CI [0.39, 0.54]. As for the other effects, neither the mindset main effect, *F*(1,194) = 0.11, *MSE* = 0.88, *p* = 0.74, $\eta_{p}^{2}$ = 0.001, [0, 0.02], nor the interaction, *F*(1,194) = 0.02, *MSE* = 0.88, *p* = 0.88, $\eta_{p}^{2}$ = 0, [0, 0.004], were significant.

#### Self-Evaluation

**FIGURE 1:**
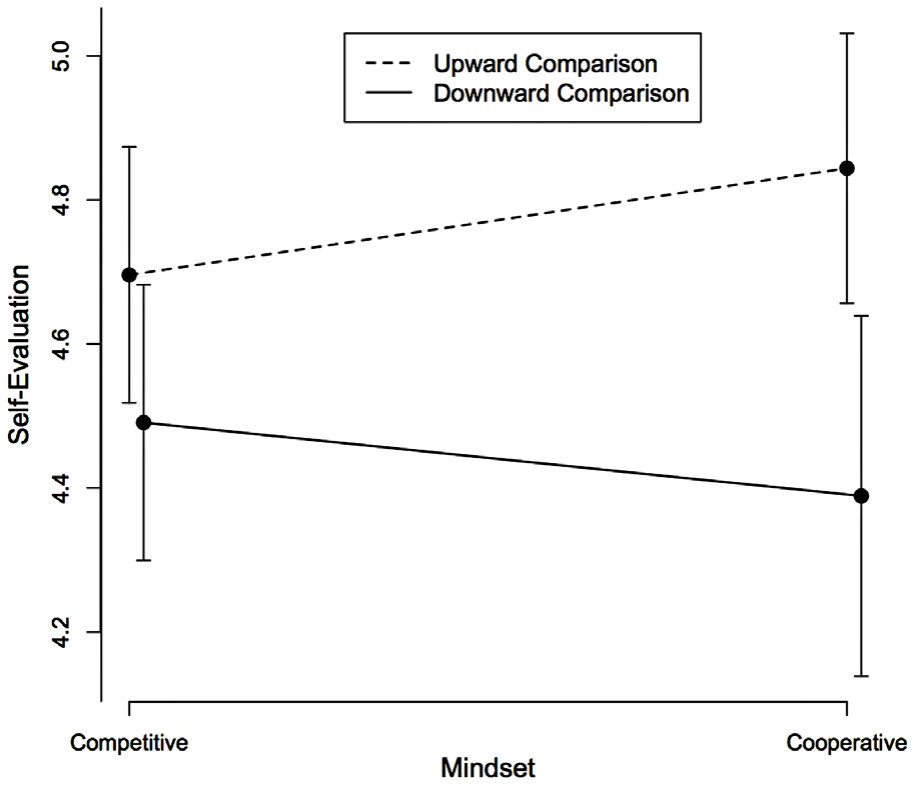
**Self-evaluation as a function of the direction of social comparison and the competitive/cooperative mindset.** Error bars represent 95% confidence intervals.

We averaged the six self-evaluation items (α = 0.76). The comparison main effect was significant, indicating that participants evaluated themselves more positively in upward comparison (*M* = 4.76, *SD* = 0.66) than in downward comparison (*M* = 4.45, *SD* = 0.73), *F*(1,194) = 10.93, *MSE* = 0.48, *p* = 0.001, $\eta_{p}^{2}$ = 0.05, [0.01, 0.11], thus revealing a generalized assimilation effect. The expected interaction did not reach the 0.05 significant level although it was in the expected direction, *F*(1,194) = 1.57, *MSE* = 0.48, *p* = 0.21, $\eta_{p}^{2}$ = 0.01, [0, 0.04]. Simple effect tests showed that under a competitive mindset, the direction of comparison had no reliable effect on self-evaluation, *F*(1,194) = 2.44, *MSE* = 0.48, *p* = 0.12, *d* = 0.30, 95% CI [–0.08, 0.67], whereas under a cooperative mindset, we found a significant assimilation effect such that participants evaluated themselves more positively in upward than in downward comparison, *F*(1,194) = 9.15, *MSE* = 0.48, *p* = 0.003, *d* = 0.66, [0.22, 1.08], (see **Figure 1**). The mindset main effect was non-significant, *F*(1,194) = 0.05, *MSE* = 0.48, *p* = 0.82, $\eta_{p}^{2}$ = 0.0, [0, 0.01].

Because some participants inaccurately reported their mindset condition at the very end of the experiment (i.e., participants who answered they were in competition while they were in cooperation or vice-versa; *N* = 38), we re-analyzed our data without them. Doing so led to only a slight (descriptive) change in statistical significance for the interaction, *F*(1,156) = 2.25, *MSE* = 0.49, *p* = 0.14, $\eta_{p}^{2}$ = 0.01, [0, 0.06]. Simple effect tests showed that under a competitive mindset, the direction of comparison had no reliable effect on self-evaluation, *F*(1,156) = 1.66, *MSE* = 0.49, *p* = 0.20, *d* = 0.27, [–0.14, 0.69], whereas under a cooperative mindset, we again found a significant assimilation effect such that participants evaluated themselves more positively in upward than in downward comparison, *F*(1,156) = 9.82, *MSE* = 0.49, *p* = 0.002, *d* = 0.75, [0.27, 1.22].

#### Similarity Ratings

This analysis first replicated the comparison main effect found by Stapel and Koomen (2005): Participants felt more similar to the upward comparison target (*M* = 3.67, *SD* = 1.46) than to the downward one (*M* = 2.60, *SD* = 1.35), *F*(1,194) = 31.12, *MSE* = 1.97, *p* < 0.001, $\eta_{p}^{2}$ = 0.14, [0.07, 0.21]. In addition, we found a marginal interaction effect, *F*(1,194) = 3.53, *MSE* = 1.97, *p* = 0.062, $\eta_{p}^{2}$ = 0.02, [0, 0.06], suggesting that the feeling of being similar to the target is more pronounced in the cooperation condition that in the competition condition. The mindset main effect was not significant, *F*(1,194) = 0.10, *MSE* = 1.97, *p* = 0.76, $\eta_{p}^{2}$ = 0, [0, 0.02].

### Discussion

Our results revealed a general assimilation effect whereby participants evaluated themselves more positively after reading about an upward than a downward comparison target (e.g., Mussweiler, 2003). More relevant to the goal of the current contribution, the critical moderation of this comparison effect by competitive/cooperative mindset was descriptively in the expected direction. Although this experiment did not seem underpowered *a priori*, we decided to test this interaction another time in Experiment 2. Instead of a second close replication, we designed a conceptual replication that improved the setting. As noted above, a supplementary goal of the second experiment was to address the problem that Stapel and Koomen’s (2005) paradigm only provided the participants with a description about the personal characteristics of the comparison other without mentioning the self, which is a limitation with regard to both the literature and real life settings. Therefore, we relied on another paradigm widely used in the social comparison literature (Muller et al., 2004; Muller and Butera, 2007; Normand and Croizet, 2013), that is a paradigm where (1) participants are provided feedback about the target and about themselves, and (2) the comparison/evaluation dimension is specific to the task (i.e., the performance in a visual task).

## Experiment 2

### Method

#### Participants

Participants were 146 undergraduate students who participated in exchange for 10 EUR. We excluded a participant who already took part in Experiment 1, and two more because they did not pay attention to the instructions (see below). Participants were randomly assigned to one of the conditions of a 2 (direction of comparison: upward vs. downward) × 2 (mindset: competition vs. cooperation) between-participants design. The final sample consisted of 143 participants (*N* = 93 women; *M*_age_ = 20.29, *SD*_age_ = 1.81).

#### Procedure

Up to four participants came to the laboratory and were assigned in pairs to one of two cubicles, each containing two computers facing each other (see Muller et al., 2004). Then participants received instructions about the attentional task (i.e., the same cueing task as in Experiment 1) they were about to perform. In this task, two white letters, an O (the target) and a Q (a distractor), appeared on a black background (one on the left side of the screen and one on the right side). For each trial, participants had to locate the target as quickly as possible. This target was preceded by a cue (a white square) that could appear on the same side or on the opposite side of the screen. After a short training, a computerized message made it clear that the coactor would perform the same task and the first recorded phase of the attentional task began.

After the first phase, instructions stated that the second phase would be completed in competition (cooperation) with the person in front of them. The instructions went on by stating that, at the end of this second phase, their results would be compared (pooled) to evaluate their performance.

After the above mindset manipulation, we manipulated the direction of comparison by using the same method as in previous experiments (Muller et al., 2004; Muller and Butera, 2007; Normand and Croizet, 2013). We told participants that the score they were about to receive was a complex calculation taking into account speed and accuracy, the perfect score being 100. Participants always received a score of 65, but the score for the coactor was 80 in the upward comparison condition and 50 in the downward comparison condition.

Next, after arguing that personal characteristics could have an impact on the scores, we asked participants four specific questions: to what extent they considered themselves *competent, efficient, fast*, and *accurate* in visual perception tasks. This was our main dependent measure, because it was at the same level of specificity as the task itself. However, to explore whether the hypothesized effects could spill over to more general measures of self-evaluation, participants also indicated the score they expected to reach in the second phase, and answered four more general questions asking whether they generally considered themselves *bright, successful, competent*, and *promising* (from 1 = *not at all* and 7 = *very*, see **Table 1B** for bivariate correlations). As mentioned previously, we excluded two participants because they told the experimenter they did not see that the traits were changing (in the software, the whole sentence stayed on the screen and only the traits changed). It is important to note that even if this measure seems similar to the main dependent variable of Experiment 1, it is quite different here in terms of specificity. In Experiment 1, these descriptive traits were at the same level of specificity as the social comparison information. In this experiment, because the social comparison information is a score regarding performance in a visual task, finding an effect on this general measure would require a generalization from visual performance to personality traits.

After being briefly reminded that they would be in competition (cooperation), participants were asked to complete the cueing task a second time. Participants then provided demographic information and were asked the same exploratory questions we used in Study 1. Again, because they did not reveal any significant effect, we will not discuss them further. Finally, three manipulation check measures were presented (“Please indicate your score,” “Please indicate the other person’s score,” and “Please indicate if you were either in competition or in cooperation with the other person”). Participants were then carefully debriefed about the goals of the experiment, thanked and dismissed.

### Results

#### Manipulation Checks

We conducted a 2 (direction of comparison: upward, downward) × 2 (mindset: competition, cooperation) × 2 (recalled score: score for the self, score for the target) ANOVA with repeated measures on the last factor. We excluded two participants from this analysis because their standardized deleted residuals were extreme (5.19 and 26.52; McClelland, 2000). Note that because these participants were outliers on the manipulation checks, we decided to exclude them from all the following analyses.

This analysis first revealed a significant main effect for the direction of social comparison, *F*(1,130) = 10152.81, *MSE* = 0.69 *p* < 0.001, $\eta_{p}^{2}$ = 0.99, [0.98, 0.99], and for the recalled score *F*(1,130) = 9.83, *MSE* = 2.59, *p* < 0.01, $\eta_{p}^{2}$ = 0.07, [0.02, 0.15]. More important, these main effects were qualified by a significant direction of comparison by recalled scores interaction, *F*(1,130) = 11852.06, *MSE* = 2.59, *p* < 0.001, $\eta_{p}^{2}$ = 0.99, [0.986, 0.991]. Simple effects analyses showed that upward comparison participants reported higher scores for the comparison target (*M*_other_ = 80.14, *SD*_other_ = 0.83) than for themselves (*M*_self_ = 64.36, *SD*_self_ = 1.90), *F*(1,130) = 6518.40, *MSE* = 2.59, *p* < 0.001, *d* = 7.05, [6.17, 7.93], while the reverse was true in the downward comparison condition (*M*_other_ = 50.15, *SD*_other_ = 0.86; *M*_self_ = 65.02, *SD*_self_ = 0.28), *F*(1,130) = 5386.23, *MSE* = 2.59, *p* < 0.001, *d* = 6.41, [5.60, 7.21].

#### Self-Evaluation in Visual Tasks

To test our main hypothesis, we averaged the four self-evaluation items (α = 0.77). We excluded one participant with a high studentized deleted residual (–4.21). The same ANOVA model used in Experiment 1 revealed a significant comparison main effect, such that participants evaluated themselves less favorably in upward comparison (*M* = 4.35, *SD* = 0.81) than in downward comparison (*M* = 4.63, *SD* = 0.71), *F*(1,136) = 6.16, *MSE* = 0.57, *p* = 0.01, $\eta_{p}^{2}$ = 0.04, [0.005, 0.11], thus revealing that here—contrary to Experiment 1—we observed a generalized contrast effect. More important, the analysis revealed the expected interaction, *F*(1,136) = 4.66, *MSE* = 0.57, *p* = 0.03, $\eta_{p}^{2}$ = 0.03, [0.001, 0.10]. It is worth mentioning that this interaction is still significant if we do not exclude the participants with a high studentized deleted residuals on the manipulation checks analysis, *F*(1,138) = 4.00, *MSE* = 0.57, *p* = 0.048, $\eta_{p}^{2}$ = 0.03, [0.0002, 0.09]. Eight participants incorrectly reported their mindset condition. When these participants are excluded, the interaction remains significant *F*(1,128) = 4.92, *MSE* = 0.59, *p* = 0.03, $\eta_{p}^{2}$ = 0.04, [0.002, 0.10].

**FIGURE 2:**
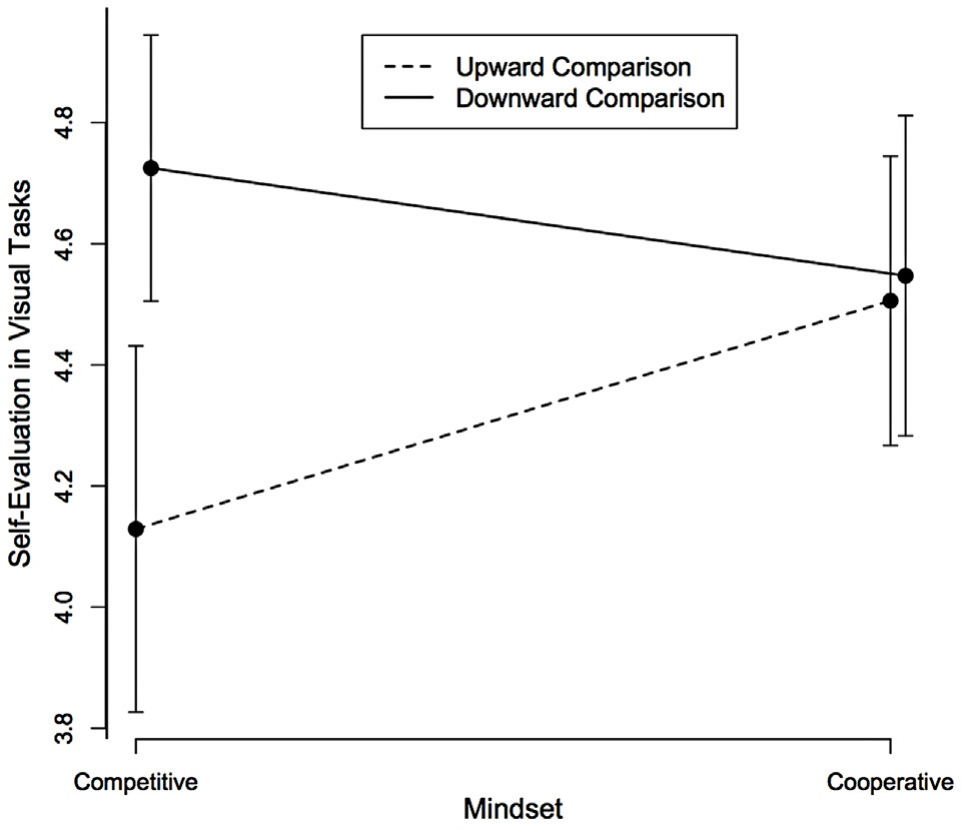
**Self-evaluation as a function of the direction of social comparison and the competitive/cooperative mindset.** Error bars represent 95% confidence intervals.

Simple effect tests showed that under a competitive mindset we found a significant contrast effect such that participants evaluated themselves less positively after an upward than after a downward comparison, *F*(1,136) = 9.56, *MSE* = 0.57, *p* = 0.002, *d* = 0.79, [0.28, 1.30], whereas under a cooperative mindset, the direction of comparison had no reliable effect on self-evaluation, *F*(1,136) = 0.06, *MSE* = 0.57, *p* = 0.81, *d* = 0.06 [–0.39, 0.50], (see **Figure 2**). The mindset main effect was not significant, *F*(1,136) = 0.60, *MSE* = 0.57, *p* = 044, $\eta_{p}^{2}$ = 0.004, [0, 0.04].

#### Expected Score

We conducted the same ANOVA on the expected score. We excluded a participant with a high studentized deleted residual (4.12). This analysis revealed a significant social comparison main effect, such that participants expected a higher score in upward comparison (*M* = 70.49, *SD* = 8.79) than in downward comparison (*M* = 63.42, *SD* = 8.24), *F*(1,131) = 22.63, *MSE* = 73.50, *p* < 0.001, $\eta_{p}^{2}$ = 0.15, [0.06, 0.24], thus revealing an assimilation effect. The mindset and the interaction effects were not reliable, *F*(1,131) = 0.89, *MSE* = 73.50, *p* = 0.35, $\eta_{p}^{2}$ = 0.007, [0, 0.05] and *F*(1,131) = 0.08, *MSE* = 73.50, *p* = 0.78, $\eta_{p}^{2}$ = 0.001, [0, 0.02], respectively.

#### General Self-Evaluation

We averaged the four items and computed a general self-evaluation index (α = 0.85). The same ANOVA revealed no reliable effects, neither for the comparison main effect, *F*(1,137) = 0.10, *MSE* = 0.90, *p* = 0.75, $\eta_{p}^{2}$ = 0.001, [0, 0.02], nor the mindset main effect, *F*(1,137) = 1.00, *MSE* = 0.90, *p* = 0.32, $\eta_{p}^{2}$ = 0.007, [0, 0.05], nor the interaction, *F*(1,137) = 0.07, *MSE* = 0.90, *p* = 0.79, $\eta_{p}^{2}$ = 0.001, [0, 0.02].

### Discussion

In this experiment, the results first revealed that participants evaluated themselves more positively after a downward comparison than after an upward comparison. Second, and more important for our purpose, this main effect was qualified by an interaction effect showing that this difference was greater under a competitive mindset than under a cooperative mindset. This interaction was significant for the self-evaluation specific to the comparison dimension (i.e., visual perception), but not for the general self-evaluation score. As we mentioned in the method section, this last measure, similar to the one used in Stapel and Koomen (2005) and our Experiment 1, was introduced for exploratory purposes because in Experiment 2 the social comparison information was clearly more specific and restrained to visual perception. We were therefore curious to see whether it would spill over to such general evaluation. It seems that it does not.

Finally, on the expected score we found, paradoxically, a general assimilation effect and not a general contrast effect as we observed in the self-evaluation measure. This might suggest that although participants’ self-evaluation suffered from upward comparison (as compared with downward comparison), it could at the same time induce a boost in motivation. Because participants feel bad, they may want to improve their performance and expect to perform better in the second phase (Johnson, 2012). This opposite effect, however, surfaced only for the comparison main effect, but not for the mindset moderation that was not significant on this measure.

## Meta-Analyses

To estimate the effect size of our predicted moderation as accurately as possible, we ran a small-scale meta-analysis (Cumming, 2012) combining our two experiments (with the initial sample size) with Studies 2 and 3 of Stapel and Koomen (2005). We chose these two experiments because they were the only ones with a design similar to ours. Their Study 3 differed from Study 2 only in that competitive/cooperative mindset was manipulated by using a scrambled sentence task.

As an effect size for the interaction effect, we computed a standardized mean difference (which, in the case of an interaction, is actually a difference of differences). Because we had no access to the *N* per condition for Stapel and Koomen’s (2005) experiments, we assumed they were equally distributed across conditions. When doing so for Study 3 one participant would be missing in one condition; we therefore randomly picked one condition for the missing participant. The raw interaction effect was computed as [(X̄*_upward_competition_* – X̄*_downward_competition_*) – (X̄*_upward_cooperation_* – X̄*_downward_cooperation_*)]/*s_p_* (where the numerator is the difference in simple effects and *s_p_* the pooled *SD*) and was subsequently corrected for sample size to get Hedges’ *g* (Borenstein, 2009). We subsequently estimated the average effect across these four experiments with a weighted random-effects model (Cumming, 2012). In a weighted model, the meta-analysis weights the contribution of each experiment based on the variance of the effect size: the contribution is stronger when the variance is low (Cumming, 2012). We chose a random model because while fixed-effect models assume that all the studies estimate the same effect size, random-effects models assume that the studies can estimate different effect sizes. Cumming (2012) recommends using random-effects models.

This meta-analysis revealed that, over those four experiments, the predicted moderation was significant, *g* = –1.49, *p* = 0.01, 95% CI [–2.67, –0.31]. It also revealed, however, that the variance across the four experiments was significant, *Q*(df = 3) = 23.06, *p* < 0.01. This suggests that the variability of the effect size was high. Because the effect sizes of our two experiments (*g* = –0.39 for Experiment 1 and *g* = –0.73 for Experiment 2) were very similar, this means that the effect sizes of Stapel and Koomen’s (2005) experiments (*g* = –2.22 for Study 2 and *g* = –2.91 for Study 3) were larger than the ones we found. To be more conservative, we therefore ran the same meta-analysis with only our two experiments to test whether the expected interaction reached significance without the surprisingly high effect sizes observed by Stapel and Koomen (2005). This analysis revealed that the effect size of the interaction was of course smaller, but still significant, *g* = –0.51, *p* = 0.02, [–0.95, –0.08]. Moreover, the variance index between our two experiments was not significant, *Q*(df = 1) = 0.69, *p* = 0.41. This last result is important, because as recently underlined by Braver et al. (2014) two experiments can differ in whether they are significant or not while being only trivially different in terms of effect size. This, Braver et al. (2014) suggest, should be seen as a better indicator of whether two studies replicate instead of their respective p values. In sum, we find evidence supporting the idea that a competitive/cooperative mindset moderates the impact of social comparison information on self-evaluation, but the effect sizes found in Stapel and Koomen’s (2005) experiments seem quite overestimated.

Although the moderation effect is the main focus of this contribution, we also conducted a meta-analysis of the comparison simple effects for the competitive and the cooperative mindset conditions. We did so for the sake of completeness and to enable a comparison with the Stapel and Koomen’s (2005) results. To conduct this analysis, we computed a standardized mean difference for the simple effects with the pooled standardized deviation of the involved means. The effect sizes were computed as (X̄*_upward_ -*X̄*_downward_*)/*s_p_* for the competitive mindset and for the cooperative mindset, respectively. Again, this index was subsequently corrected for sample size to get Hedges’ *g*. We applied the same steps as before: we report first a meta-analysis including our results and Stapel and Koomen’s (2005) and then a meta-analysis including only our results. For the competitive mindset, the meta-analysis revealed that, across the four experiments, the contrast effect was marginal, *g* = –0.65, *p* = 0.07, [–1.35, –0.04]. The variance index among these four experiments was significant, *Q*(df = 3) = 23.53, *p* < 0.001. When relying only on our results, the meta-analysis revealed that this contrast effect was not significant, *g* = –0.25, *p* = 0.56, [–1.34, 0.85]. The variance index was again significant, *Q*(df = 1) = 11.84, *p* < 0.001, indicating, unsurprisingly (because one was clearly significant while the other was not), a significant variability between our two experiments. For the cooperative mindset, the meta-analysis revealed that, across the four experiments, the assimilation effect was significant, *g* = 0.81, *p* = 0.03, [0.08, 1.53]. The variance index among these four experiments was significant, *Q*(df = 3) = 18.827, *p* < 0.001. When relying only on our results, the meta-analysis revealed that this assimilation effect was not significant, *g* = 0.30, *p* = 0.40, [–0.39, 0.98]. Also, unsurprisingly, the variance index was again significant, *Q*(df = 1) = 4.92, *p* = 0.03.

## General Discussion

Social comparison takes place either in competitive or in cooperative settings. It is therefore crucial to uncover whether these settings moderate the impact of social comparison on self-evaluation. In Experiment 1, our results revealed that the predicted moderation was weaker than the one reported by Stapel and Koomen (2005), whereas the results of Experiment 2 revealed a significant moderation. Importantly, however, a small-scale meta-analysis on our two experiments confirmed that a competitive/cooperative mindset moderates the impact of social comparison on self-evaluation.

These two experiments represent an important contribution for several reasons. First, to the best of our knowledge, no experiments have directly or conceptually replicated the moderating role of competitive/cooperative settings (Stapel and Koomen, 2005), notwithstanding the theoretical and applied importance of this effect. The results of our two experiments and our meta-analysis showed that not only did we succeed in replicating Stapel and Koomen (2005), but we also managed to reinforce this demonstration by adding a quite different paradigm (i.e., Experiment 2).

Second, these results contribute to some extent to the Inclusion/Exclusion model (Schwarz and Bless, 2007). This model indeed predicts that a competitive/cooperative mindset should moderate the effect of social comparison, but, as Schwarz and Bless (2007) noted, only Stapel and Koomen (2005) tested this moderation. Given the doubt that can be raised about these studies, the current work therefore extends the scope of the Inclusion/Exclusion model to such an important structural variable.

Third, we believe that the demonstration of such a contextual moderation (i.e., being in competition or in cooperation) is important in the social comparison literature, because—in contrast with many experimental operationalizations used as moderators (e.g., walking away from or toward a screen, Fayant et al., 2011, or searching for dissimilarities vs. similarities between two drawings, Mussweiler, 2001)—the manipulation of competition/cooperation shows that the effect of social comparison on self-evaluation can be moderated by a highly ecological variable. In line with this idea, a look at the work citing Stapel and Koomen’s (2005) article shows that it has been cited in a great deal of applied work (for instance, in organizational psychology). This illustrates that the moderation effect studied in the present research is also an important contribution to understanding social comparison effects that occur outside the laboratory, where competition and cooperation are defining features of social interaction.

Fourth, Experiment 2 also shows, for the first time, that the moderation of the effects of social comparison on self-evaluation by a competitive/cooperative mindset can be found even when participants are provided with feedback on a specific dimension, and not only with such general information as having good social skills (see the present Experiment 1, and Stapel and Koomen, 2005). This is a theoretical addition that has important practical implications because in real life settings people often compare specific and precise feedback that they receive with feedback that others receive, such as at school where students have access to their grades and to others’ grades (Blanton et al., 1999).

Fifth, these experiments also contribute to research on the effect of social comparison on cognitive processes (e.g., Huguet et al., 1999; Muller and Butera, 2007; Normand and Croizet, 2013). Indeed, the self-evaluation threat model assumes that upward comparison represents a threat to self-evaluation (i.e., a contrast effect) and that, in turn, this threat consumes attention normally allocated to the task at hand (Muller et al., 2004; Muller and Butera, 2007). The current experiments suggest that previous work conducted in this area might have found results in line with this assumption because the experimental settings were construed as competitive.

Finally, the present results also have implications for educational practices, because they suggest that a cooperative setting can decrease harm to self-evaluation when students compare themselves to other better-off students. For example, by encouraging a cooperative mindset, teachers might have a critical role in shaping how other children’s grades can influence one’s self-evaluation, making an upward comparison with other children beneficial to self-evaluation (Ames, 1981). Of course, the other side of the coin might be that the self-evaluation of better-off children could be dragged down by worse-off children.

Interestingly, although the meta-analysis showed that our two experiments displayed the same effect size for the moderation effect (i.e., the same interaction), the pattern of their cell means is somewhat different (see **Figures 1** and **2**). Indeed, as explained in great detail in Rosnow and Rosenthal (1991), two patterns of cell means (the four means in a 2 × 2 design) can look different although their interactions are identical and only their main effects are different. This is precisely what happens with our two experiments where the interaction was similar, but the two experiments had different social comparison main effects. Indeed, in Experiment 1 the comparison main effect amounted to an assimilation effect, while in Experiment 2, it amounted to a contrast effect. This also explains why different simple effects emerged as significant in the two experiments (an assimilation effect in cooperation in Experiment 1 and a contrast effect in competition in Experiment 2) and why they failed to reach significance in the meta-analysis of our two experiments.

It is important to recall that the focus of the current piece was to test whether a competitive/cooperative mindset *moderates* the impact of social comparison on self-evaluation. We did not have specific predictions regarding the main effect of social comparison and the resulting simple effects. Actually, several factors have been found to moderate the impact of social comparison information (see Mussweiler, 2007 or Bless and Schwarz, 2010, for reviews). Hence, it is difficult to predict the direction of the main effect of social comparison by itself. Inspecting the differences between our two experiments, we can only speculate about the factors responsible for the asymmetry of the main effects. One explanation might rely on the specific differences in methods in the two experiments: In Experiment 1 we provided only information about the comparison target, whereas in Experiment 2 we provided information about both the comparison target and the self. Maybe providing information about the self and the comparison target leads to focus more on the self than providing information about the comparison target only. Another explanation might be that in our two experiments we used measures with different levels of specificity. Hence, it could be, for instance, that a general measure is more likely to elicit assimilation effects. Because this was not the focus of the current piece, our interest clearly residing in the moderation (i.e., the interaction) and not the main effects, these are only speculations and future experiments should test this hypothesis within the same experimental design. These specifications notwithstanding, the interaction effect we found confirms that, as suggested by the Inclusion/Exclusion model (Schwarz and Bless, 2007), it is critical to pay attention to the way people construe the situations in which comparison takes place.

It is noteworthy that Experiment 1 revealed a marginal interaction on similarity ratings. This might suggest that a similarity focus could be an interesting mediator of the competitive/cooperative mindset effect. According to the selective accessibly model, a search for differences between the self and the comparison target would elicit a contrast effect, while a search for similarities would elicit an assimilation effect (Mussweiler, 2003). A search for similarities could be the underlying process that enables the inclusion of the representation of the comparison target into the self-representation. Future studies could explore this issue by determining the role of a focus on similarities in this phenomenon.

Although future experiments are needed to explore these questions further, the current work demonstrates the moderating role of a competitive/cooperative mindset on the effects of social comparison on self-evaluation. Hence, the experiments reported here suggest that when researchers and practitioners care about the effect that social comparison information can have on self-evaluation, it is crucial to take into account whether the context promotes a competitive or a cooperative mindset.

## Conflict of Interest Statement

The authors declare that the research was conducted in the absence of any commercial or financial relationships that could be construed as a potential conflict of interest.

## Footnotes

1 When participants are not provided with feedback, scholars sometime used the terms “positive” and “negative” comparison (e.g., Stapel and Koomen, 2005). Instead, we used “upward” and “downward” to be consistent all along the manuscript.
