# Supplementary material for: A mindset of competition versus cooperation moderates the impact of social comparison on self-evaluation
Source: Front Psychol. 2015 Sep 3;6:1337. doi: 10.3389/fpsyg.2015.01337 (PMC4558468; doi:10.3389/fpsyg.2015.01337)
Supplement: Supplementary file 3 [file Table_1.DOCX]

Table 1

*Means and standard-deviations as a function of Mindset and Direction of Social Comparison*

|  | Competition | | |  | Cooperation | | |  |  |  |  |
| --- | --- | --- | --- | --- | --- | --- | --- | --- | --- | --- | --- |
|  | Upward Comparison  (*n* = 57) |  | Downward Comparison  (*n* = 55) |  | Upward Comparison  (*n* = 47) | Downward Comparison  (*n* = 39) | |  |  | Effect sizes (η_p_^2^) |  |
| Variables | M (SD) |  | M (SD) |  | M (SD) |  | M (SD) |  | Social Comparison | Mindset | Interaction |
| Self-evaluation^a^ | 4.70 (0.67) |  | 4.49 (0.71) |  | 4.84 (0.64) |  | 4.39 (0.77) |  | .05** | 0 | .008 |
| Similarity ratings^b^ | 3.47 (1.48) |  | 2.73 (1.42) |  | 3.91 (1.41) |  | 2.41 (1.25) |  | .138*** | 0 | .018† |

^a^ Self-evaluation is composed of six 7-point scale items (from 1 = *not at all* and 7 = *very*). A higher score reflects more positive evaluations.

^b^ Similarity ratings is composed of one 7-point scale item (from 1 = *not at all* and 7 = *very much*).

*** *p* < .001, ** *p <* .01, * *p <* .05, † *p* < .10.

Table 2

*Means and standard-deviations as a function of Mindset and Direction of Social Comparison*

|  | Competition | | |  | Cooperation | | |  |  |  |  |
| --- | --- | --- | --- | --- | --- | --- | --- | --- | --- | --- | --- |
|  | Upward Comparison  (*n* = 31) |  | Downward Comparison  (*n* = 31) |  | Upward Comparison  (*n* = 42) | Downward Comparison  (*n* = 37) | |  |  | Effect sizes (η_p_^2^) |  |
| Variables | M (SD) |  | M (SD) |  | M (SD) |  | M (SD) |  | Social Comparison | Mindset | Interaction |
| Self-evaluation in visual tasks ^a^ | 4.13 (0.82) |  | 4.73 (0.59) |  | 4.51 (0.77) |  | 4.55 (0.79) |  | .043* ^d^ | .004 | .033* |
| Expected score ^b^ | 71.55 (9.09) |  | 64.00 (8.92) |  | 69.71 (8.58) |  | 63.00 (7.82) |  | .147*** ^e^ | .007 | .001 |
| General self-evaluation ^c^ | 4.40 (0.86) |  | 4.40 (1.07) |  | 4.61 (0.89) |  | 4.51 (0.97) |  | .001 | .007 | .001 |

^a^ Self-evaluation in visual tasks is composed of four 7-point scale items (from 1 = *not at all* and 7 = *very*). A higher score reflects more positive evaluations.

^b^ The score participants expected in the second task (from 1 to 100).

^c^ General self-evaluation is composed of four 7-point scale items (from 1 = *not at all* and 7 = *very*). A higher score reflects more positive evaluations.

^d^ One outlier on this analysis.

^e^ One outlier on this analysis. Five missing values on this analysis.

*** *p* < .001, ** *p <* .01, * *p <* .05, † *p* < .10.
